# Cortical response to proprioceptive stimulation in primary orthostatic tremor – a magnetoencephalography study

**DOI:** 10.1016/j.cnp.2025.04.002

**Published:** 2025-05-02

**Authors:** Karolina af Edholm, Mikkel C. Vinding, Christoph Pfeiffer, Anders Svenningsson, Erik Fransén, Mathias Sundgren, Henrik Sjöström, Niklas Edvall, Daniel Lundqvist, Josefine Waldthaler

**Affiliations:** a Department of Clinical Sciences, Karolinska Institutet, Campus Danderyd Hospital, Stockholm, Sweden; b Department of Clinical Neuroscience, Karolinska Institutet, Stockholm, Sweden; c Department of Psychology, University of Copenhagen, Copenhagen, Denmark; d School of Electrical Engineering and Computer Science, KTH Royal Institute of Technology, Stockholm, Sweden; e Digital Futures, KTH Royal Institute of Technology, Stockholm, Sweden; f Science for Life Laboratory, KTH Royal Institute of Technology, Stockholm, Sweden; g Department of Neurology, Karolinska University Hospital, Stockholm, Sweden; h Centre for Neurology, Academic Specialist Centre, Stockholm, Sweden

**Keywords:** Orthostatic tremor, Magnetoencephalography, Proprioception

## Abstract

**Objective:**

Patients with primary orthostatic tremor (OT) are affected by a strong feeling of instability. Our aim was to investigate whether cortical response to proprioceptive information is altered in OT compared with healthy control subjects (HC) using magnetoencephalography (MEG).

**Methods:**

Fifteen OT patients and 15 HCs were examined using whole-head MEG. Proprioceptive stimulation was evoked by passive movements of the right index finger and right foot. Time-frequency responses were extracted in the frequency range of 8–30 Hz. The MEG sensor with the highest amplitude change time-locked to the proprioceptive stimulus was used to calculate the amplitude of event-related desynchronization (ERD) and subsequent event-related re-synchronization (ERS).

**Results:**

Finger stimulation induced reliable ERD and ERS, as expected, in both OT patients and HC. Foot stimulation induced weaker ERS in both groups. Cluster-based permutation tests did not reveal any significant differences between the OT and HC groups.

**Discussion:**

We did not find evidence supporting a generally altered cortical response to proprioceptive stimulation in patients with OT.

**Significance:**

This is the first study on proprioceptive cortical processing in OT. The results do not support the interpretation that the characteristic intense feeling of instability in OT is linked to altered cortical response to proprioceptive information.

## Highlights

• We investigated cortical response to proprioceptive stimulation in orthostatic tremor.
• Passive movements of the finger and foot were used for proprioceptive stimulation.
• Event-related desynchronization and subsequent synchronization was normal.

## 1 Introduction

Primary orthostatic tremor (OT) is a rare neurological disorder in which fast synchronised tremor occurs in the leg and trunk muscles when activated during standing (Bhatia et al., 2018). The tremor frequency is 13–18 Hz (Piboolnurak et al., 2005), which is significantly higher than that of other tremor disorders, such as Parkinson’s disease (4–7 Hz) and essential tremor (<12 Hz) (Deuschl et al., 2022). In each affected individual, the frequency is rather constant over time (Ganos et al., 2016). In addition to tremors, OT patients are strongly affected by an intense feeling of unsteadiness while standing, which progresses over time, eventually resulting in the need for walking aids (Feil et al., 2015, Ganos et al., 2016, Gerschlager et al., 2004, Hassan et al., 2016). OT is a neurodegenerative disorder with a gradual onset, usually starting in the 6th decade and with a slight predominance in women. There are indications of mild cognitive and cerebellar impairment as well as a high prevalence of psychiatric comorbidities (Benito-Leon et al., 2016b). Common tremor medications, such as beta-blockers and antiepileptic drugs, are often less effective than in other tremor disorders. Bilateral deep brain stimulation in the ventro-intermediate nuclei of the thalami or zona incerta can be partially effective, at least initially (Boogers et al., 2022, Gilmore et al., 2019, Guridi et al., 2008, Merola et al., 2017; Espay et al., 2008).

The aetiology of OT remains unknown. It has been hypothesised to be initiated by either a supraspinal central oscillator or a disruption in the cerebello-thalamo-cortical network involved in postural control (Bacsi et al., 2005, Deuschl et al., 2001, Gallea et al., 2016, Muthuraman et al., 2013, Schoberl et al., 2017, Wu et al., 2001). However, this does not fully explain the characteristic intense feeling of unsteadiness in OT, which does not prevail in other tremor disorders involving the cerebello-thalamo-cortical network, such as essential tremor. It has been proposed that pathological integration of proprioceptive information induces this subjective feeling of unsteadiness (Fung et al., 2001, Mohwald et al., 2020, Schoberl et al., 2017, Wuehr et al., 2018). We hypothesised that the integration could be affected by the conflicting proprioception from the fast tremor activity affecting afferent proprioceptive signals from the legs or by pathological changes impeding the integration of proprioceptive information.

Imaging studies using different modalities have revealed discrete volumetric and functional changes in the cerebellum, brainstem, thalamus, and sensorimotor cortex in OT (Benito-Leon et al., 2016a, Benito-Leon et al., 2019, Gallea et al., 2016, Schoberl et al., 2017, Wills et al., 1996). Given the evidence of alterations at the cortical level, studying cortical activity could shed further light on the integration of sensory information in OT patients. Only a few neurophysiological studies have used electroencephalography (EEG) in OT, and no previous studies have used magnetoencephalography (MEG). No remarkable pathologies have been associated with OT in routine EEG (Hellman et al., 2021), but increased oscillating activity in the OT frequency range has been observed in the central midline and around the primary motor cortex in patients with OT during standing (McManis and Sharbrough, 1993, Muthuraman et al., 2013). While MEG detects the same underlying neural activity as EEG, the magnetic fields generated by neural activity are less affected by the surrounding tissue than the electric fields, giving MEG a higher spatial resolution than EEG (Cheyne, 2013).

In the cortex, movement or sensory stimuli are associated with cortical beta-band activity (15–30 Hz), specifically, event-related desynchronization (ERD) during the active sensorimotor state. ERD is a decrease in beta-band oscillations detectable over the sensorimotor cortex up to approximately one second before a voluntary movement is initiated and prevails during the movement. ERD is followed by event-related synchronization (ERS) or “beta rebound”, which is a transient increase in beta-band activity (Cheyne, 2013). Voluntary and passive movements are associated with coherent ERD and ERS in primary motor cortex and somatosensory cortex, whereby the ERS component has been linked to the integration of afferent proprioceptive information (Parkkonen et al., 2015). Healthy individuals present equal ERS to active and passive movement, but no response when proprioceptive afferent information is inhibited by peripheral nerve block (Cassim et al., 2001) indicating that ERS is involved in the integration of proprioceptive afferent feedback necessary for motor control (Piitulainen et al., 2013, Engel and Fries, 2010 Apr). This is further supported by the fact that in neurodegenerative disorders such as Parkinson’s disease, where reduced proprioception is considered to be related to supratentorial degeneration, ERS is reduced. Divergences in ERD and ERS in response to passive movement reveal a pathological integration of proprioceptive afferent feedback at the cortical level (Mujunen et al., 2022, Vinding et al., 2019; Vinding et al., 2020). Thus, studying cortical signals in response to passive movements may broaden our understanding of the mechanisms underlying OT.

Here, we present our results from the first MEG study in OT with proprioceptive stimulation of the index finger and foot in a cohort of 15 OT patients and 15 healthy matched controls. We hypothesised that reduced ERS to proprioceptive stimulation in OT patients compared with healthy controls is an effect of changes in the integration of proprioceptive information. OT patients were examined in a seated position to ensure that the results were not influenced by afferent sensory information from tremor activity.

## 2 Method

### 2.1 Participants

Fifteen patients and 15 healthy controls (HC) were enrolled in the study between March and November 2023. Patients with OT were recruited from the Neurology Clinic of the Danderyd Hospital in Stockholm, Sweden. The inclusion criteria in the OT group were EMG-confirmed orthostatic tremor of 13–18 Hz according to the consensus statement of the classification of primary orthostatic tremor (Bhatia et al., 2018), and a Montreal Cognitive Assessment (MoCA) score of 22 or higher. Exclusion criteria in both the OT and HC groups were any pathological findings during neurological examination by a neurologist (KE, JW), indicating other neurological disorders or peripheral neuropathy. 20 healthy controls were initially recruited from a pool of individuals who had previously participated in other MEG studies. One healthy control was excluded because of previously undiagnosed ataxia discovered during the clinical examination. Four healthy males were excluded from further analysis to match the groups by age and sex. The study was approved by the Swedish ethical review authority (Etikprövningsmyndigheten, ref.no 2019-01490 and 2022-01802-02), and all participants provided written informed consent. The study was conducted in accordance with the Good Clinical Practice guidelines and the principles of the Declaration of Helsinki.

### 2.2 Procedure

MEG recordings were performed at the Swedish National Facility for Magnetoencephalography (NatMEG) at the Karolinska Institute, Sweden in 2023. Participants were allowed to take any regular medication. Patients were treated with clonazepam (n = 3), gabapentin (n = 3), gabapentin + clonazepam (n = 1), propranolol (n = 3), and gabapentin + propranolol (n = 1), and four patients did not use any tremor-modifying medication. For patients using clonazepam, we ensured that the medication intake was more than one hour before MEG registration in all cases.

Prior to MEG, participants were examined using the Unified Parkinson’s Disease Rating Scale (UPDRS) Part 3, MoCA Scale, Hospital Anxiety and Depression Scale (HADS), and the 10-item Orthostatic Tremor Severity and Disability Scale (OT-10). If patients had already completed the scales during the last six months, these results were used instead.

### 2.3 Proprioceptive stimulation

**Fig. 1:**
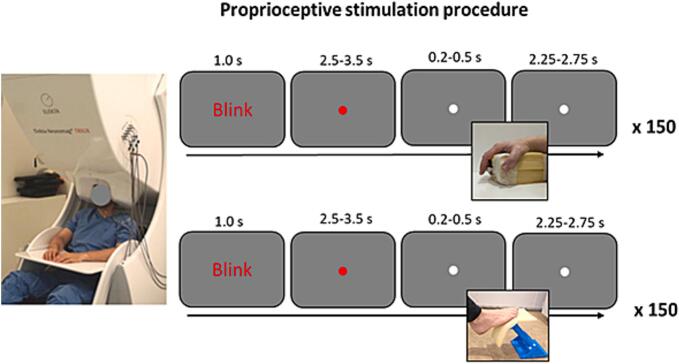
Proprioceptive stimulation procedure. Participants were seated in the MEG system with HPI and EMG electrodes attached. Two custom-made MEG-compatible pneumatic movement actuators were used to induce passive movement of the right index finger and of the right foot. Finger and foot stimulation was repeated 150 times, respectively. Each stimulation started with the instruction to blink, followed by a visual stimulus in the shape of a dot that changed colour from red to white. The participants were instructed to blink while the dot was red and to keep their eyes open while the dot was white. The passive movement was initiated during the period of open eyes. (For interpretation of the references to colour in this figure legend, the reader is referred to the web version of this article.)

The proprioceptive stimulation of the upper and lower limbs was performed similarly to Piitulainen et al. (2015) and Vinding et al. (2019), consisting of passive movements of the index finger of the right hand (the dominant hand in all participants except one) and the right foot evoked by custom-made MEG-compatible pneumatic movement actuators, respectively (Fig. 1).

The movement actuators were driven by pneumatic artificial muscles that contract when pressurised and expand during the pressure release, resulting in linear motion. The flow of pressurised air to the device was manipulated using valves outside the magnetically shielded room using Presentation software (v. 18.3; Neurobehavioral Systems, Inc., https://www.neurobs.com).

To induce passive movement stimulation of the index finger of the right hand, the participant's hand was relaxed on a table with the index finger resting on the actuator (Fig. 1). Participants were explicitly instructed to relax with their eyes open, avoid any voluntary movement, and focus their gaze on a fixation dot at the centre of a screen in front of them. To avoid blinking during the induced movements, every trial started with the instruction “blinka” (English “blink”) on the screen, which was followed by a visual stimulus where the dot on the centre of the screen changed colour from red to white (Fig. 1). The visual stimulus was presented after a random time interval of 2500 to 3500 ms, upon which the passive movement was induced after an additional interval randomized between 200 and 500 ms. The actuator moved the distal phalanx of the index finger, inducing an extension and flexion of the fingertip of 0.5 cm, lasting approximately 200 ms (movement range 10-15°, angular velocity 50-75°/s). A new trial began after a randomised interstimulus interval between 2250 and 2750 ms.

The second proprioceptive stimulus was the passive movement of the right foot. The leg of the patient was relaxed, resting on steady cushions with the forefoot resting on the actuator and ankle at approximately 90°. The actuator moved the foot by inducing plantar flexion and dorsiflexion of the ankle, resulting in a movement of the distal part of the foot of 1 cm in 200 ms (movement range 2.5–3.5°, angular velocity 12.5–17.5°/s). This was repeated in the same way as the description for the finger. Note that in contrast to the passive movements of the foot induced by Piitulainen et al. (Piitulainen et al., 2015), our device moved not only the big toe but also the entire forefoot (Fig. 1).

All participants confirmed that they could sense the movement of the finger or foot during a series of ten test stimulations prior to the recording. The participants were able to see the moving finger and foot during the stimulation, but were asked to focus their gaze at the screen. In total, 150 trials were recorded for each condition. Before and after the proprioceptive stimulations, resting state with eyes open was recorded for 5 min. Alertness was checked by the examiners between MEG recordings using the Karolinska Sleepiness Scale (KSS). A snack and/or drink was offered if participants felt tired to avoid falling asleep during measurements.

### 2.4 MEG data acquisition

The MEG data were recorded with a whole-head 306-channel TRIUX MEG system (MEGIN Oy, formerly Elekta Neuromag) in a two-layer magnetically shielded room (MSR). The sensors are arranged in triplets consisting of one magnetometer and two orthogonally arranged planar gradiometers at 102 locations distributed around the participant’s head. Recordings were performed according to the protocol of Vinding and colleagues (Vinding et al.,2019). The sampling frequency was 1000 Hz. An online 0.1 Hz high-pass filter and 330 Hz low-pass filter were used. Head position was continuously recorded using head-position indicator coils (HPI) to detect head movements. The HPI was digitised with a Polhems Fast Track Motion tracker during preparation. Electrooculograms (EOG) and electrocardiograms (ECG) are used to capture eye muscle and heartbeat artifacts. Electromyography (EMG) with bipolar Ag/AgC electrodes was used to detect possible voluntary muscle activation of the examined index finger (finger flexor and extensor) and foot (tibialis anterior and lateral head of gastrocnemius). Correct positioning of the EMG electrodes was achieved by palpating muscle movements while the participants actively performed the respective movements that would later be passively induced by the movement actuator. An accelerometer was placed on the middle phalanx of the right index finger and on the back of the right forefoot to track movements along three orthogonal axes. The continuous time course of the accelerometer was sampled together with the MEG data.

### 2.5 Data processing

MEG data were pre-processed with temporal signal space separation using MaxFilter software (Taulu and Simola, 2006) with a buffer length of 10 s and a cut-off correlation coefficient of 0.98 to suppress artifacts from outside the scanner helmet. The data processing script is available on GitHub (https://github.com/JoWld/proprioception_OT). Independent component analysis (ICA) was performed using the *fastica* algorithm in MNE-Python (Gramfort et al., 2013), and components related to eye blinks and heartbeats, identified by correlating them with measured EOG and ECG peaks, were removed. The data were subsequently partitioned into epochs of 1.5 s before passive stimulations to 3.5 s after stimulation. Trials with extreme jump artifacts based on a min-to-max peak range exceeding 10 pT for the magnetometer and 2000 fT/cm for gradiometers were rejected.

Epochs containing passive stimulation were further analysed with MATLAB R2022b (MathWorks Inc., Natick, MA) using the FieldTrip toolbox (Oostenveld et al., 2011). Accelerometer data were used to confirm the successful execution of passive movements and to exclude trials with accidental active movements outside the stimulation period (0–500 ms from stimulus onset). The accelerometer data were filtered with a band-pass filter between 1 and 195  Hz before averaging the three orthogonal channels by calculating the Euclidian norm and z-score normalizing the average. After data cleaning, 138 ± 3 (OT: 121–150; HC: 137–150) trials in finger registration and 135 ± 18 (OT: 96–150; HC: 100–150) trials in foot registration remained per subject for further analysis.

The remaining trial epochs were averaged for each condition and for each participant. The average response within the subject was subtracted from each trial to improve sensitivity to non-phase-locked responses (Kalcher and Pfurtscheller, 1995). We defined a region of interest (ROI), including 16 (finger) and 23 (foot) orthogonal gradiometer pairs over the left sensorimotor cortex (Supplementary Table 1 and Supplementary Fig. 1). Given the cortical representation of the foot along the interhemispheric fissure, the ROI for foot stimulation was extended to encompass the channels closer to the midline. The gradiometer pair within the respective ROI with the highest mean value of the obtained event-related field in the interval 50–110 ms after stimulus onset was selected to represent the sensory-motor response and used for further analysis (Supplementary Material), as described in detail by Vinding and colleagues (Vinding et al., 2019). We obtained the event-related responses by time–frequency decomposition using wavelets with a width of five cycles in the time window starting 1.25  s before movement onset and ending 2.5  s after on all frequencies from 2–40  Hz in steps of 1 Hz. The gradiometer pairs were then combined to obtain a single time–frequency representation for each pair location. Finally, the time–frequency representation in the 8–30 Hz frequency range was extracted from the selected peak-channel pair before the data were log-transformed and baseline-corrected by subtracting the average power in the baseline interval 1250 s to 200 ms before stimulus onset from the power values in each frequency bin. The resulting time–frequency response, expressed as the log-transformed absolute power change in dB from baseline, was used for statistical comparison between the groups.

The EMG data were filtered using a discrete Fourier transform filter to suppress 50 Hz line noise and cut into epochs corresponding to the time windows of interest for the MEG. After rectifying the signal, the EMG epochs were averaged for each session for each subject. For further comparison, we calculated the power spectral density of the non-rectified EMG signals using a fast Fourier transform after applying a Hann window.

### 2.6 Statistical analysis

Group differences in demographics and clinical characteristics were tested using t-tests and chi-square tests. The time–frequency representations for finger and foot stimulation were compared between the groups using cluster-based permutation tests with 1000 random permutations (Maris and Oostenveld, 2007), a nonparametric test that controls for multiple comparisons across the time–frequency window of interest (Maris and Oostenveld, 2007). Each time–frequency point was compared between groups using a two-tailed *t*-test, and clusters of significant differences were defined by summing the t-values of neighbouring time–frequency points with a p-value < 0.05 (two-tailed). The sum of the clusters of t-values was compared to a random distribution of cluster values calculated in the same way from random permutations of data assigned to the groups using a Monte Carlo simulation (n = 1000). Clusters in which the total sum of t-values was on the edges of the permutation distribution beyond the critical alpha of 0.05 were considered significant.

Spearman correlations were used to explore the relationship between ERD, expressed as relative change from baseline and extracted for the period between 100 ms and 500 ms after stimulus onset, and clinical scores (age, disease duration, MoCA, and OT-10). Given the exploratory nature of this analysis, we did not correct for multiple comparisons.

Potential group differences due to voluntary muscle activation during passively induced movements (0 to 500 ms after stimulus) were checked by comparing the EMG signals during the movement with EMG signals from a baseline period (−500 ms to 0 ms) using cluster-based permutation tests with 1000 random permutations.

## 3 Results

### 3.1 Clinical parameters

In total, 15 OT patients and 15 HC fulfilled all the inclusion criteria and none of the exclusion criteria and were included in the analysis. Participants’ variables are presented in Table 1. There were no significant group differences between the OT patients and HC in terms of age (*t*-test, p = 0.378) or female-to-male ratio (chi-squared test, p = 0.292). The OT patients had a median symptom duration of 11 years (range 3–26 years) and a median orthostatic tremor frequency of 15.3 Hz (range 13.5–17 Hz).

**Table 1:** Clinical properties and variables of OT patients and healthy controls. Age, disease duration and tremor frequency are reported as median and range, p-values are calculated with two-tailed *t*-test. Female and male distribution is reported as a ratio, p-value is calculated with Chi square test. Scores of MoCA, UPDRS III, OT-10 and HADS are reported as mean and standard deviation, p-values are calculated with two-tailed *t*-test.

	Orthostatic Tremor patients (n = 15)	Healthy Controls (N = 15)	p-value
Age (years)	70 (50–81)	74 (51–80)	0,378
Female:Male	11:4	9:6	0,929
MoCa	27,4 ± 2.0	26.0 ± 1.9	0.049*
UPDRS III	2.4 ± 2.0	0.8 ± 1.1	0.012*
OT-10	26.2 ± 9.6	0.47 ± 1.4	<0.001*
HADS	8.9 ± 5.2	3.4 ± 4.0	0.004*
Disease duration (years)	11 (3–26)		
Tremor frequency (Hz)	15.3 (13.5–17)		

Symptoms of OT were generally high in the OT group, with a mean score of 26.2 ± 9.6 on the OT-10 rating scale (0–50). OT patients tended to have a slightly higher average MoCA score (27.4 ± 1.8) than HC (26.0 ± 1.9; *t*-test, p = 0.049; t = 2.05) and a higher HADS score (8.79 ± 5.2) than HC (3.40 ± 4.0; *t*-test, p = 0.004; t = 3.12). There was also a significant difference between the scores of the UPDRS part III (*t*-test, p = 0.012; t = 2.70), although both groups scored low (2.4 ± 2.0 among OT patients versus 0.8 ± 1.1 among HC). In the OT group, postural and kinetic tremors of the upper extremities were the most common symptoms in the UPDRS part III.

### 3.2 Time-frequency response

**Fig. 2:**
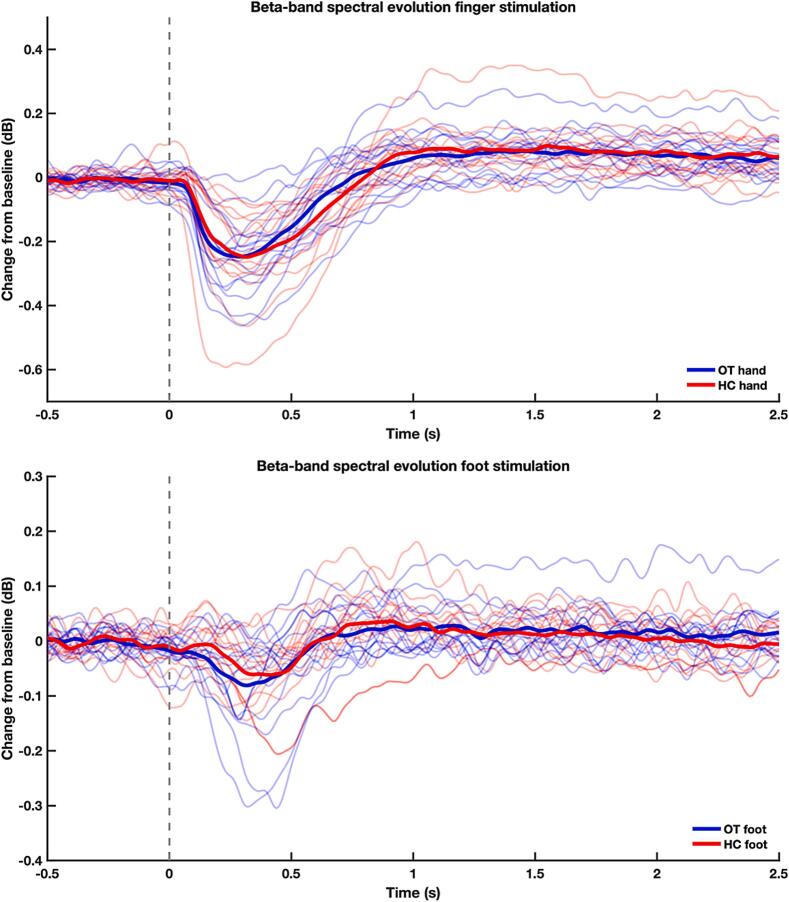
Baseline-corrected log-normalised time–frequency responses at the peak channel showing the temporal evolution of the relative change of alpha/beta band (8–30 Hz) oscillatory activity in response to proprioceptive stimulation of the finger (upper row) and foot (lower row). Stimulation onset at time = 0 is represented by the dashed vertical line. The average of the OT group is presented in red, HC group in blue. Semi-transparent lines represent the averaged responses per subject. NB different scaling of the y axis for finger and foot stimulation. (For interpretation of the references to colour in this figure legend, the reader is referred to the web version of this article.)

**Fig. 3:**
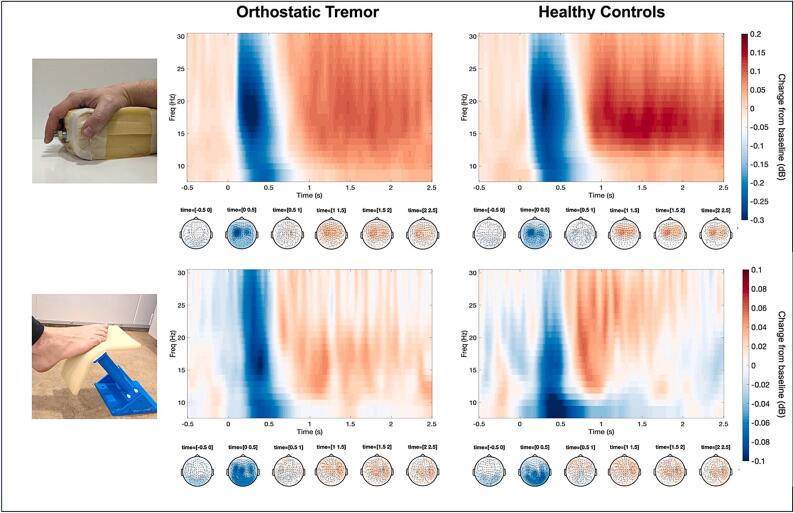
Beta response to proprioceptive stimulation (8–30 Hz). Peak-channel time–frequency representations (TFR) and topographic maps of the cortical response to proprioceptive stimulation of the finger (upper row) and foot (lower row). Passive movement onset at time = 0. NB different scaling for finger and foot stimulation.

Finger stimulation caused ERS and ERD in both groups (Fig. 2, upper row), similar in size to that expected in healthy individuals in previous studies using the same stimulation device (Vinding et al., 2019). Stimulation of the foot, however, only induced a weaker ERD (Fig. 2, lower row). Upon visual inspection, reliable ERS or *rebound* appeared to be absent in both the groups. However, this impression was not confirmed by post-hoc one-sample cluster-based permutation t-tests, resulting in evidence for a difference between the pre-selected time window of the ERS response (500 ms to 1000 ms) and the baseline time window (−500 ms to 0 ms) in both the HC (p = 0.013) and OT groups (p = 0.027), defined by a single cluster starting 710 ms (HC), and 760 ms (Yague et al.) after stimulus onset, respectively. Please see Supplementary Figs. 2 and 3 for individual responses from all participants. The time–frequency representations and topographic maps are shown in Fig. 3.

Cluster-based permutation tests comparing the evoked responses across the entire trial duration between the HC and OT groups did not show any significant differences in the time–frequency responses in the alpha/beta band (8–30 Hz) to the proprioceptive stimulation of the right index finger (p = 0.639) or the right foot (p = 0.239). Likewise, the mean peak-to-peak amplitude did not differ between groups (finger stimulation: OT 0.617* 1.00E−11 ± 0.382*1.00E−11, HC 0.603*1.00E−11 ± 0.455*1.00E−11, t = 0.55, p = 0.9; foot stimulation: OT 0.236*1.00E−11 ± 0.114*1.00E−11, HC: 0.214*1.00E−11 ± 0.103*1.00E−11), t = 0.09, p = 0.6).

The magnitude of ERS and ERD may depend on the spectral power in the mu-band during the baseline period leading up to the movement. We therefore compared absolute power spectral density in the time-window from 1.25  s to 0.2  s before stimulation using the same statistical approach based on cluster-based permutation test as described for our main comparison. Using 1000 random permutations and a two-tailed cluster-forming threshold of p = 0.05, no clusters were identified at any point in this analysis window, indicating no contiguous time points or frequencies within the mu range where the groups differed even at an uncorrected threshold.

We repeated the analysis of the time–frequency responses, excluding the four participants with OT who had taken clonazepam prior to the MEG recordings, because benzodiazepines have been shown to increase event-related desynchronization in the beta band. This exclusion resulted in no changes in the group-wise comparisons described above (see Supplementary Fig. 4 for the group-averaged ERS/ERD).

An exploratory correlation analysis of individual ERD values (expressed as change from baseline) did not yield any evidence for associations of age, disease duration, OT 10, MDS-UPDRS III, or MoCA score with ERD after finger or foot stimulation (see Supplementary Table 2 for complete results).

Visual inspection of individual EMG signals and additional cluster-based permutation tests on EMG data did not show evidence of voluntary muscle activation or group differences in the time window of the time–frequency analysis.

## 4 Discussion

This is the first study in OT using MEG to test the hypothesis that disturbed response to proprioceptive information contributes to the pathophysiology of the disease. The exceptionally high temporal resolution of MEG compared to other functional neuroimaging modalities such as fMRI or PET makes it suitable for investigating altered cortical reactions to proprioceptive sensory stimuli, one of the proposed mechanisms underlying OT symptoms.

The steady progression of prominent instability sensations in OT that occurs only in standing, without convincing signs of peripheral neuropathy, vestibular dysfunction, visual impairment, or very distinct cerebellar atrophy or ataxia, emphasizes the hypothesis that proprioceptive afferent information is somehow compromised at the cortical level. This may be the result of disturbing proprioceptive information from fast tremor activity or by direct neurodegenerative changes in the sensorimotor cortex. Therefore, our study investigated whether there are differences in cortical proprioceptive processing measured as ERS and ERD in the sensorimotor mu rhythm for passive movement between OT patients and healthy controls. Beta-ERD is commonly interpreted as a state of heightened sensitivity to efferent and afferent information in the motor cortex, allowing for subsequent effective movement execution and proprioceptive integration (Jenkinson and Brown, 2011). However, no difference between the OT patients and the control group could be detected. Since stimulation of the index finger induced appropriate beta-ERS and ERD in both controls and OT patients, technical flaws could be excluded.

The stimulation of the foot induced a weaker, although significant, ERS and ERD in both groups compared to the finger stimulation and previous studies (Mujunen et al., 2022, Piitulainen et al., 2015), indicating that our method of foot stimulation might have been insufficient for this purpose. Considering the low responses, a subsequent group comparison was not deemed informative. Nonetheless, the results from the stimulation of the index finger provide evidence that the response to proprioceptive information in patients with OT may be unaffected, at least for the upper extremities. Based on our results, we cannot confirm that a disintegration of proprioceptive information at the cortical level is a contributing factor to the intense feeling of unsteadiness described by patients with OT.

Since our method of foot stimulation did not evoke a prominent beta rebound in either controls or patients, we cannot exclude the effects of proprioceptive stimulation of the foot on cortical response in OT. The representation of the foot in the sensory cortex lies in the most medial part of the postcentral gyrus, where the lower extremity is represented from distal to proximal in the mediolateral direction with the toes in the anteroposterior direction (Akselrod et al., 2017). The area of stimulation is thus close to the scalp and should be available for MEG registration; however, the representation of the foot is less selective and smaller than the representation of the hand, which could influence the level of beta power (Zhao et al., 2019). In contrast to the study by Piitulainen et al., in which passive stimulation of the big toe resulted in a significant beta band response (Piitulainen et al., 2015), we investigated passive stimulation of the whole foot to better simulate movements induced by commonly involved muscles in the OT, such as the tibialis anterior and gastrocnemius. However, in contrast to the study by Mujunen et al., where ankle rotation also was used and resulted in a significant beta band response (Mujunen et al. 2022), our stimulation started with a plantarflexion followed by a dorsiflexion, and had a smaller movement range and slower angular velocity, factors that may explain the less prominent beta band response in our study.

A limitation of our study is that the number of participants was relatively low, which is a recurrent problem when studying rare diseases such as OT. There is a high interindividual time–frequency variability, which has been shown to be even higher in the lower limb (Mujunen et al. 2022), that further complicates the reliability of the small sample size. The low statistical power due to the small sample size might have masked smaller effects of OT on proprioceptive processing. Peripheral neuropathy could also be a confounding factor, especially in an elderly population. Even mild peripheral neuropathy has been correlated to alterations in beta band response to proprioceptive stimulation, like stronger beta-suppression, stronger beta power in sensorimotor area 1, and weaker beta rebound (Mujunen et al., 2025). None of our participants were presenting signs of peripheral neuropathy by clinical examination, but electroneurography could be considered useful to fully exclude subclinical neuropathy. The correlation between behavioral measures of sensory perception and ERS/ERD on MEG could be a possible clue to understand the Beta-rebound as a measure of proprioceptive integration and should be further clarified in future studies.

The use of GABA-potentiating drugs may also influence cortical responses. Four patients with OT were administered clonazepam regularly (0.5–1 mg up to three times a day), which could have affected the results in these patients. However, the results did not change significantly when patients taking clonazepam were excluded (Supplementary Fig. 4). GABA-enhancing drugs, such as benzodiazepines, augment background beta-band activity, thereby potentially facilitating the ERD component while leaving the ERS unaffected (Cheyne, 2013). Thus, it is unlikely that the use of clonazepam reduced the ERD response to proprioceptive stimulation in our study. Cortical response may be affected by other medications and psychostimulants, as well as by drowsiness. In the current study, patients were allowed to continue their regular medications to facilitate their traveling to the test site. Examining patients in an off-medication state would be a valuable extension of our study.

Our study was performed with participants in a seated position, and symptoms of OT occur while standing, which explains why patients did not present with tremors during the registrations. This allowed us to study the cortical integration of proprioceptive information without disturbing tremor activity. The patients were fully relaxed when examined in our study, but a light isometric activation of the examined limb could also be a way of enhancing the beta band response in future studies (Giangrande et al., 2024). Another step would be to study cortical integration in the presence of tremors. Conventional whole-head MEG measurements are mainly possible in sitting or supine positions, but on-scalp MEG will facilitate measurements in the standing position and could be used to reveal further information on the pathology of OT. Investigating the source level of beta activity and expanding the analysis to other frequency bands could also be a future prospect.

## 5 Conclusion

In this study, we found no evidence for differences in cortical responses to proprioceptive stimulation, measured as event-related desynchronization and synchronization, between patients with orthostatic tremor and healthy controls. Thus, our results do not support the interpretation that the characteristic intense feeling of instability in OT patients could be explained as a generally altered cortical response of proprioceptive information. However, technical aspects of the foot stimulation may be improved before this mechanism can be excluded.

## Ethics statements

The study was approved by the Swedish Ethical Review Authority (Etikprövningsmyndigheten, ref.no 2019–01490 and 2022–01802-02). All participants provided written informed consent in accordance with the Declaration of Helsinki.

## Declaration of Competing Interest

The authors declare that they have no known competing financial interests or personal relationships that could have appeared to influence the work reported in this paper.

## Appendix A Supplementary data

Supplementary Figure 1 Channels included in the Regions of Interest (ROI). Colours display how often channels were selected within each group.

Supplementary Figure 2 Individual responses for each healthy control participant. Finger stimulation is depicted in light orange, foot stimulation in dark red, respectively.

Supplementary Figure 3 Individual responses for each OT patient. Finger stimulation is depicted in blue, foot stimulation in turquoise, respectively. Asterix (*) indicates patients who took clonazepam prior to the MEG recording.

Supplementary Figure 4 Beta band evolution without the four patients using clonazepam (N = 11). NB difference in scale for finger and foot stimulation.
