# Supplementary figures and images for: Cortical response to proprioceptive stimulation in primary orthostatic tremor – a magnetoencephalography study
Source: Clin Neurophysiol Pract. 2025 May 2;10:159–66. doi: 10.1016/j.cnp.2025.04.002 (PMC12747180; doi:10.1016/j.cnp.2025.04.002)

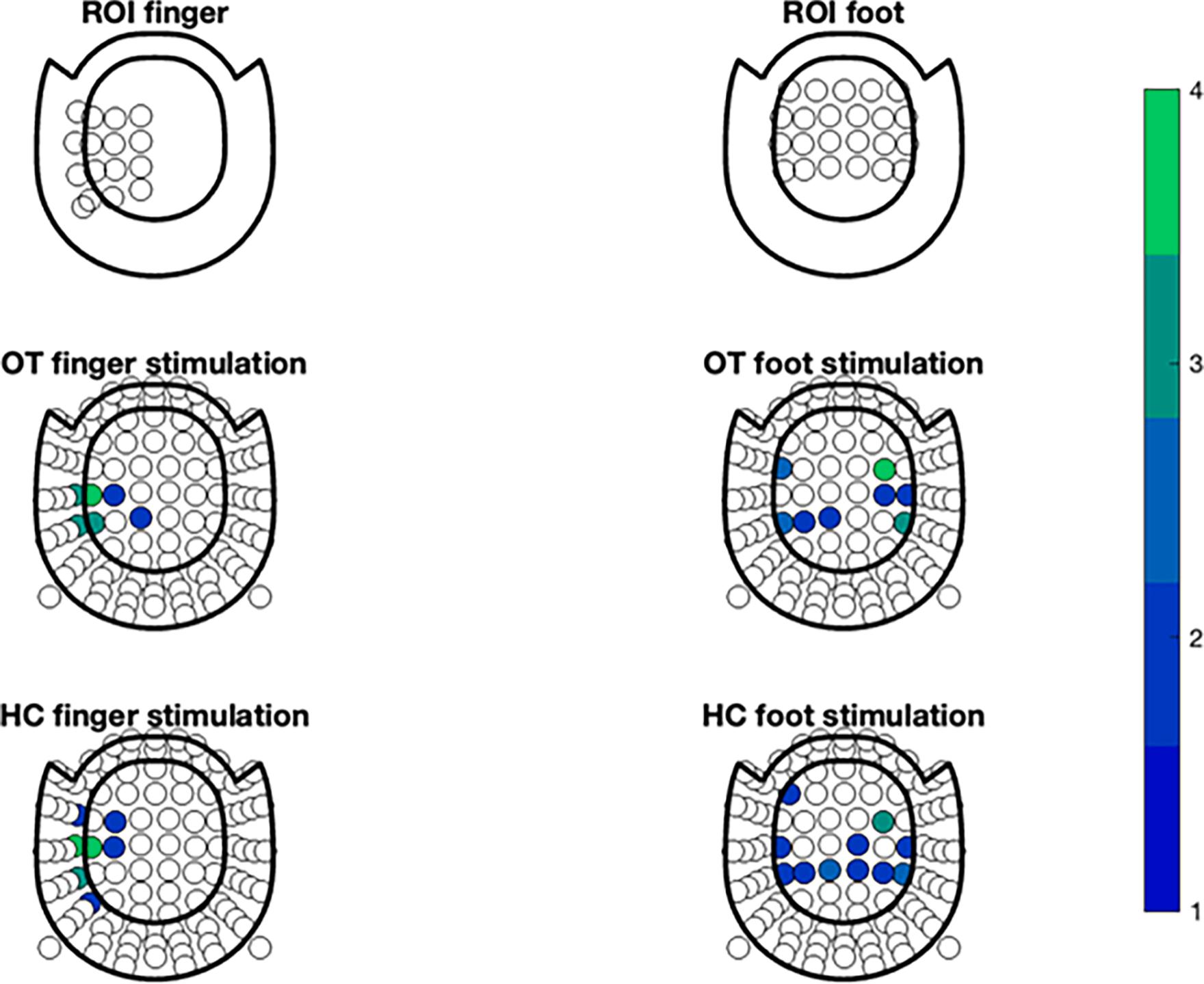

Supplement: Supplementary Figure 1 — Channels included in the Regions of Interest (ROI). Colours display how often channels were selected within each group. [file mmc1.jpg]

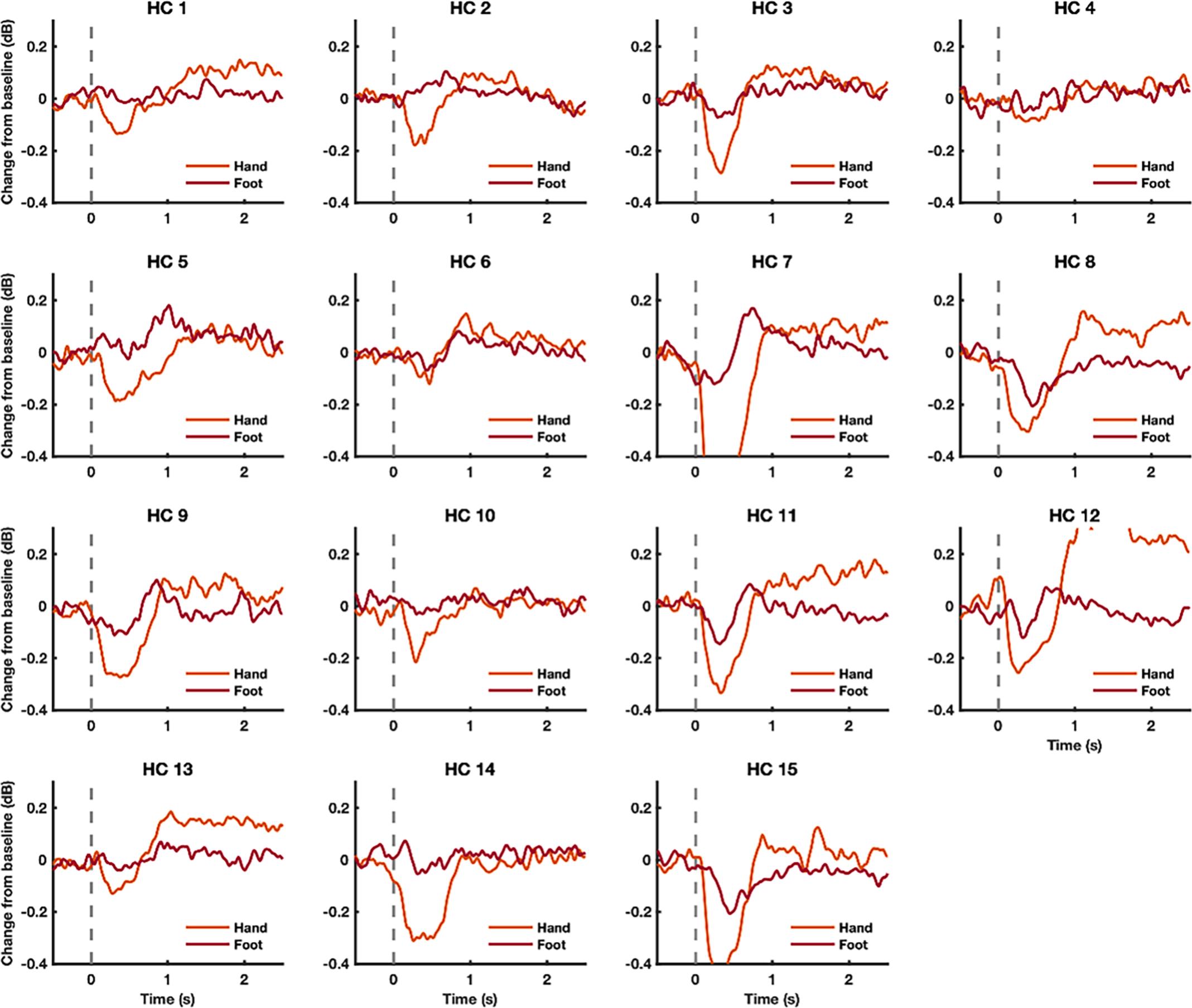

Supplement: Supplementary Figure 2 — Individual responses for each healthy control participant. Finger stimulation is depicted in light orange, foot stimulation in dark red, respectively. [file mmc2.jpg]

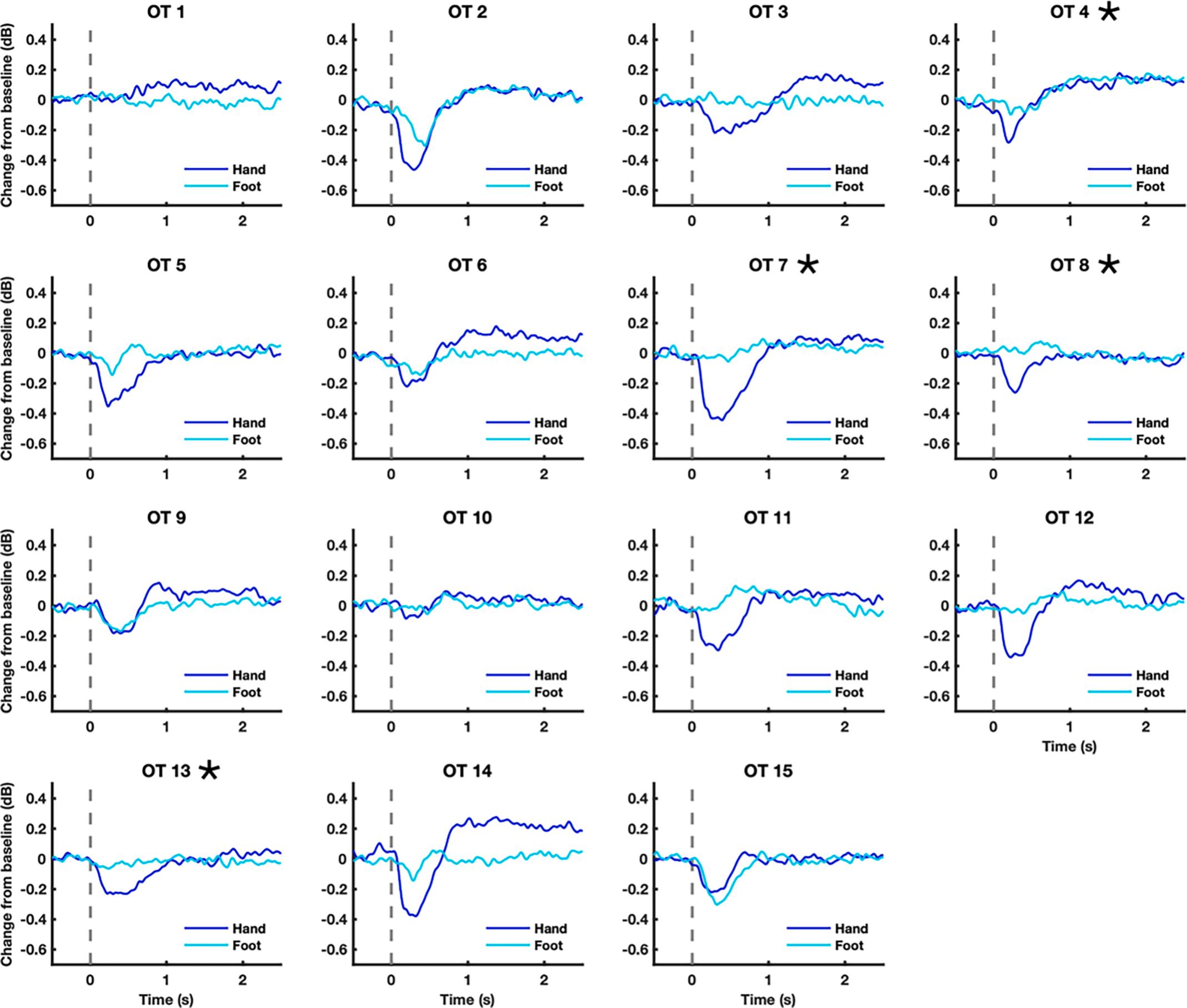

Supplement: Supplementary Figure 3 — Individual responses for each OT patient. Finger stimulation is depicted in blue, foot stimulation in turquoise, respectively. Asterix (*) indicates patients who took clonazepam prior to the MEG recording. [file mmc3.jpg]

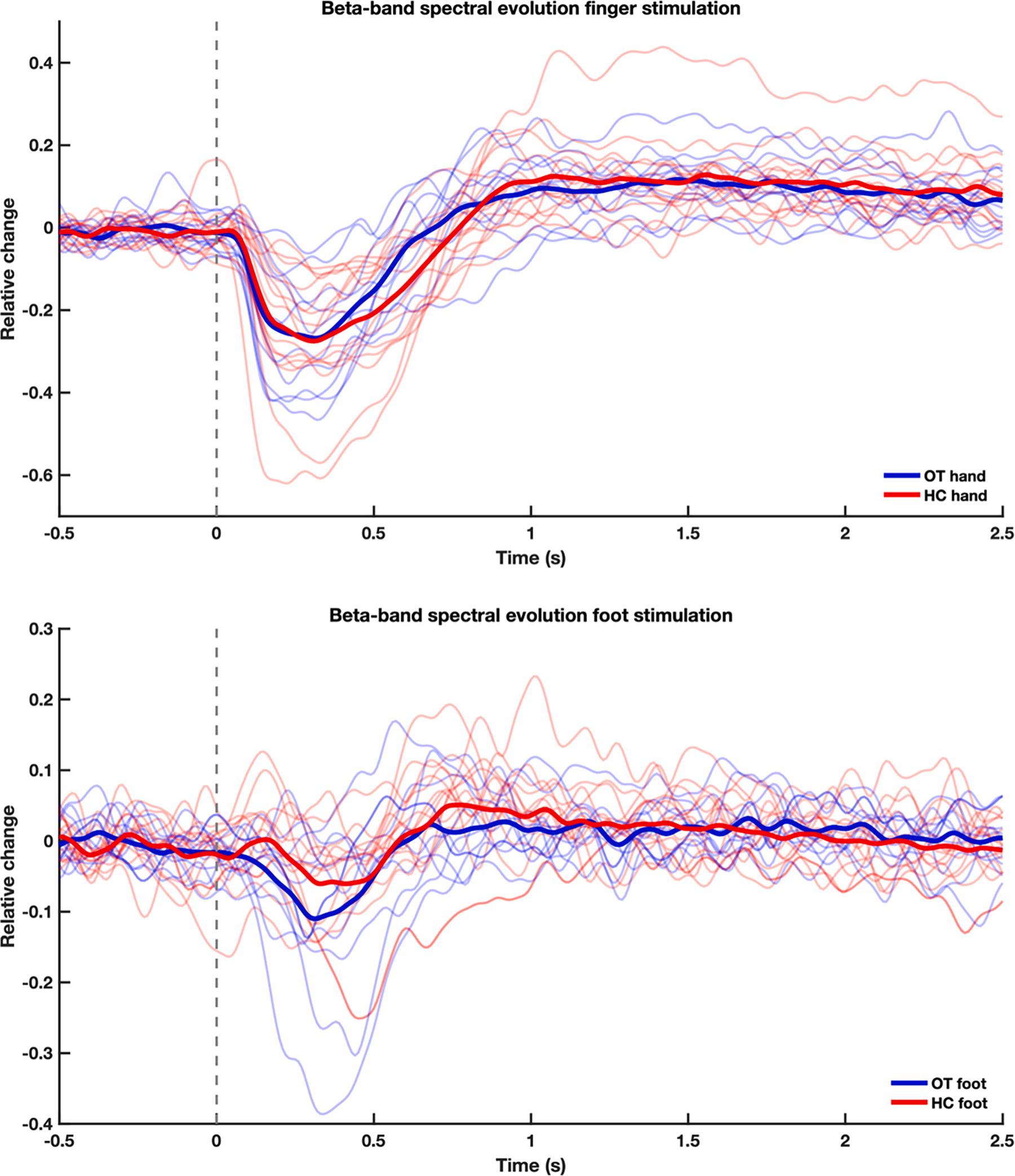

Supplement: Supplementary Figure 4 — Beta band evolution without the four patients using clonazepam (N = 11). NB difference in scale for finger and foot stimulation. [file mmc4.jpg]
