# Supplementary material for: Cortical response to proprioceptive stimulation in primary orthostatic tremor – a magnetoencephalography study
Source: Clin Neurophysiol Pract. 2025 May 2;10:159–66. doi: 10.1016/j.cnp.2025.04.002 (PMC12747180; doi:10.1016/j.cnp.2025.04.002)
Supplement: Supplementary Data 5 [file mmc5.docx]

Supplementary Table 1.

| Finger stimulation | | | Foot stimulation | | |
| --- | --- | --- | --- | --- | --- |
| gradiometer pair finger | **N OT** | **N HC** | **gradiometer pair** | **N OT** | **N HC** |
| MEG0222+0223 | 0 | 1 | MEG0332+0333 | 0 | 1 |
| MEG0232+0233 | 3 | 4 | MEG0412+0413 | 2 | 0 |
| MEG0412+0413 | 0 | 0 | MEG0422+0423 | 0 | 0 |
| MEG0422+0423 | 0 | 1 | MEG0432+0433 | 0 | 0 |
| MEG0432+0433 | 1 | 1 | MEG0442+0443 | 0 | 1 |
| MEG0442+0443 | 4 | 4 | MEG0622+0623 | 0 | 0 |
| MEG0632+0633 | 0 | 0 | MEG0632+0633 | 0 | 0 |
| MEG0712+0713 | 0 | 0 | MEG0642+0643 | 0 | 0 |
| MEG0742+0743 | 1 | 0 | MEG0712+0713 | 0 | 0 |
| MEG1622+1623 | 3 | 3 | MEG0722+0723 | 0 | 1 |
| MEG1632+1633 | 0 | 1 | MEG0732+0733 | 0 | 1 |
| MEG1642+1643 | 0 | 0 | MEG0742+0743 | 1 | 2 |
| MEG1812+1813 | 3 | 0 | MEG1032+1033 | 0 | 0 |
| MEG1822+1823 | 0 | 0 | MEG1042+1043 | 0 | 0 |
| MEG1832+1833 | 0 | 0 | MEG1112+1113 | 4 | 3 |
| MEG1842+1843 | 0 | 0 | MEG1122+1123 | 0 | 0 |
|  |  |  | MEG1132+1133 | 1 | 1 |
|  |  |  | MEG1142+1143 | 1 | 0 |
|  |  |  | MEG1242+1243 | 0 | 0 |
|  |  |  | MEG1812+1813 | 2 | 1 |
|  |  |  | MEG1822+1823 | 1 | 1 |
|  |  |  | MEG2212+2213 | 0 | 1 |
|  |  |  | MEG2222+2223 | 3 | 2 |
