# Supplementary material for: Cortical response to proprioceptive stimulation in primary orthostatic tremor – a magnetoencephalography study
Source: Clin Neurophysiol Pract. 2025 May 2;10:159–66. doi: 10.1016/j.cnp.2025.04.002 (PMC12747180; doi:10.1016/j.cnp.2025.04.002)
Supplement: Supplementary Data 6 [file mmc6.docx]

Supplementary Table 2.

|  | ERD finger |  | ERD foot |  |
| --- | --- | --- | --- | --- |
|  | r | p-value | r | p-value |
| Age (years) | 0.02 | 0.932 | 0.12 | 0.932 |
| Disease duration (years) | 0.14 | 0.619 | -0.02 | 0.619 |
| OT10 | 0.11 | 0.699 | 0.24 | 0.699 |
| UPDRS III | 0.22 | 0.424 | 0.06 | 0.424 |
| MoCA | -0.13 | 0.657 | -0.16 | 0.657 |
| ERD foot mu-band | 0.08 | 0.771 |  |  |
